# Autofluorescence spectroscopy and multispectral autofluorescence microscopy for characterization of lupus nephritis in renal tissues

**DOI:** 10.1038/s41598-026-47510-w

**Published:** 2026-07-02

**Authors:** Pramila Thapa, Vishesh Dubey, Azeem Ahmad, Kristin Andreassen Fenton, Dalip Singh Mehta, Balpreet Singh Ahluwalia

**Affiliations:** 1 Bio and Green Photonics Lab, Department of Physics, Indian Institute of Technology, New Delhi, Delhi 110016 India; 2 Department of Physics and Technology, UiT the Arctic University of Norway, Tromsø, Norway; 3 RNA and Molecular Pathology Research Group, Department of Medical Biology, Faculty of Health Sciences, UiT the Arctic University of Norway, Tromsø, Norway

**Keywords:** Autofluorescence, Lupus nephritis, Spectroscopy, Multispectral microscopy, Biological techniques, Optics and photonics

## Abstract

The study investigates lupus nephritis in label-free mode using simultaneous autofluorescence spectroscopy and multispectral autofluorescence microscopy. Lupus nephritis (LN) is an autoimmune disease with proteinuria as the most typical symptom and indicator of renal damage and disease activity. This study includes the development and application of a micro-spectro-endoscope on the renal tissues of murine LN, covering three stages, advanced end-stage (proteinuric), early onset disease (antibody-positive), and healthy with no disease manifestations (young). The micro-spectro-endoscope exploits the autofluorescence (AF) at two excitation wavelengths, 365 and 405 nm, to probe endogenous autofluorescence activity. Multispectral autofluorescence microscopy performed using a 365 nm light emitting diode (LED) collected information from three spectral bands, 440–490 nm, 500–560 nm, and 600–650 nm, respectively. Three regions, medulla, medulla-cortex, and cortex, were investigated using autofluorescence microscopy, and the maximum autofluorescence was recorded in the cortex region. Acquiring simultaneous AF spectroscopy and AF microscopy provided molecular and morphological information from the same tissue location. Simultaneous autofluorescence microscopy and spectra of kidney tissues from proteinuric, antibody-positive, and young mice were recorded with a micro-spectro-endoscope by exciting at 405 nm LED. A significant change in the peak of the autofluorescence spectrum for proteinuric, antibody-positive, and young mice was observed, with peaks around ~ 480 nm, ~ 493 nm, and ~ 532 nm, respectively. The alternation in the autofluorescence peak is likely due to the variations of nicotinamide adenine dinucleotide and flavin adenine nucleotide fluorophores during the progression of the disease. An additional peak around ~ 616 nm is also observed in antibody-positive tissue, showing the presence of lipofuscin, which is absent in young and proteinuric tissues, indicating the possibility of increasing lysosomal activity and oxidative stress.

**Supplementary Information:**

The online version contains supplementary material available at 10.1038/s41598-026-47510-w.

## Introduction

Autofluorescence (AF), referred to as native fluorescence, is a widespread phenomenon found in biological tissues owing to the abundance of intrinsic biomolecules that possess inherent fluorescence properties^1^. The AF emission is mainly linked to the structural and functional properties of biomolecules. The AF is with the intrinsic biomarkers, such as nicotinamide adenine dinucleotide (NADH), flavin adenine dinucleotide (FAD), porphyrins, collagens, etc., present in many biological specimens^1–3^. The subtle alterations at the molecular level can be investigated using this technique, as these fluorophores actively participate in diverse cellular and tissue functions, such as metabolic reactions within the cells. The intrinsic AF profile can also be altered due to variations in the tissue’s absorption and scattering mechanisms^4^. Oxyhemoglobin and deoxyhemoglobin within the tissues are attributed to the absorption phenomena^5,6^. The scattering is mainly associated with the refractive index heterogeneity in the tissue^7,8^. Consequently, alterations in the light-tissue interaction open up the opportunity to measure observable changes and use this information as an indication of the disease progression within the tissues. Variations in the intrinsic fluorophores, collagen levels, nuclear size distribution, epithelial thickness, and other morphological and molecular factors can induce shifts in the absorption and scattering signals. These variations cause a change in the AF spectra exhibited by the diseased tissues, exploiting them as a biomarker^4^. Cell and tissue conditions during normal or abnormal processes in any disease progression may lead to alterations in the quantity and arrangement of these endogenous fluorophores^1,2^. Furthermore, this may result in changes in their surroundings’ chemical and physical characteristics^9^. The distinct attributes of AF may serve as sensitive indicators, enabling the detection of subtle alterations throughout disease progression. This capability may aid in diagnosing diseases like cancer and its progression, hyperplasia, and dysplasia, etc., across different stages^4^. The AF technique has been extensively utilized in cancer research for diagnostic and classification purposes, with numerous studies conducted in this area^4,10–12^.

The AF technique has also been used to probe kidney dysfunctions^13,14^. Systemic lupus erythematosus (SLE) is an autoimmune disease, and when it primarily affects the kidneys, it is known as lupus nephritis (LN). The most common manifestation of LN is proteinuria. Proteinuria is a biological indicator of renal damage and disease activity, and it is counted as a hallmark of SLE disease activity^15^. The progression of LN leads to chronic kidney disease (CKD) caused by interstitial and renal tubule fibrosis^16,17^. The increase in CKD cases worldwide, along with the rise in end-stage renal failure requiring renal replacement therapy, makes it an epidemic in the upcoming years^18^. The CKDs refer to a range of conditions that affect the kidneys’ structure and function^19^. The progression of CKD from LN is mainly due to the increase in antinuclear antibodies (ANAs)^20^. ANAs are characteristic markers of autoimmune connective tissue disorders. These antibodies are specific to various elements within the cell nucleus, encompassing proteins, DNA, RNA, and complexes formed by nucleic acids and proteins^20^. Standard diagnostic procedures such as ANA blood tests, urinalysis, tissue biopsy, and MRI scans are employed to identify autoimmune conditions. Specifically, LN diagnosis involves blood and urine tests, including a 24-hour collection and a kidney biopsy^21,22^. Ultimately, confirmation and assessment of disease progression in LN primarily rely on the insights provided by the kidney biopsy, which is a very lengthy procedure^17^.

Optical methods such as AF microscopy and AF spectroscopy are emerging as non-invasive, non-contact techniques that offer real-time, in vivo disease diagnosis and screening capabilities^10^. Endoscopy can be used to diagnose autoimmune diseases, especially those affecting the gastrointestinal tract^21,23^. In addition to endoscopy, micro-endoscopy gives an idea about microscopic structures and changes within the body. Various micro-endoscopic systems have been developed for disease detection and screening^12^. These developed micro-endoscopes often use a large number of optical components, making them highly complex systems^24,25^. Furthermore, only endoscopy has been used for diagnostic purposes of LN or any other autoimmune diseases^26^.

Here, we developed a micro-spectro-endoscope (MSE) for in vitro tissue analysis of LN, integrating two complementary modalities,microscopy and spectroscopy,simultaneously within a single platform. Although renal parenchyma is not readily accessible using standard clinical endoscopy, this study aims to demonstrate the technical feasibility and analytical advantage of combining AF microscopy and spectroscopy in a unified micro-endoscopic system for renal tissue assessment. The micro-spectro-endoscope incorporates a graded refractive index (GRIN) rod lens specifically designed for micro-endoscopic applications. GRIN rod lenses exhibit a radial variation in refractive index, making them particularly suitable for miniaturized optical systems and high-resolution micro-endoscopy. The simultaneous utilization of microscopy and spectroscopy within the MSE provides complementary information on morphological changes and molecular alterations within a single measurement procedure. This concurrent approach enhances the system’s sensitivity and diagnostic capability, as spectroscopy enables detection of molecular-level transitions alongside visual assessment of morphological variations.

In the present proof-of-concept implementation, a 2 mm diameter GRIN rod lens was employed to perform ex vivo micro-endoscopic measurements in MRL renal tissue. The experimental concept is not limited to this specific geometry and can be extended to needle-based or fiber-based micro-endoscopic configurations, supporting potential future minimally invasive in vivo applications. The current study focused on employing the developed MSE in the AF technique to investigate LN progression and its various pathological stages, including proteinuria. Two excitation wavelengths, 365 nm and 405 nm, were used to probe tissue AF characteristics associated with different endogenous fluorophores. Multispectral AF microscopy was performed using 365 nm excitation with three emission bands (450–490 nm, 510–560 nm, and 600–650 nm). Simultaneous AF spectroscopy and AF microscopy were conducted using 405 nm excitation. The rationale for selecting these excitation wavelengths was to target AF signals arising from distinct renal biomarkers. The 365 nm excitation predominantly probes NADH and FAD-related compounds, while 405 nm excitation additionally enhances sensitivity to porphyrins alongside NADH and FAD^1,2^. To our knowledge, this is the first investigation demonstrating integrated AF microscopy and spectroscopy using a micro-spectro-endoscope to detect stage-dependent changes during LN progression.

## Materials and methods

### Experimental setup

**Fig. 1:**
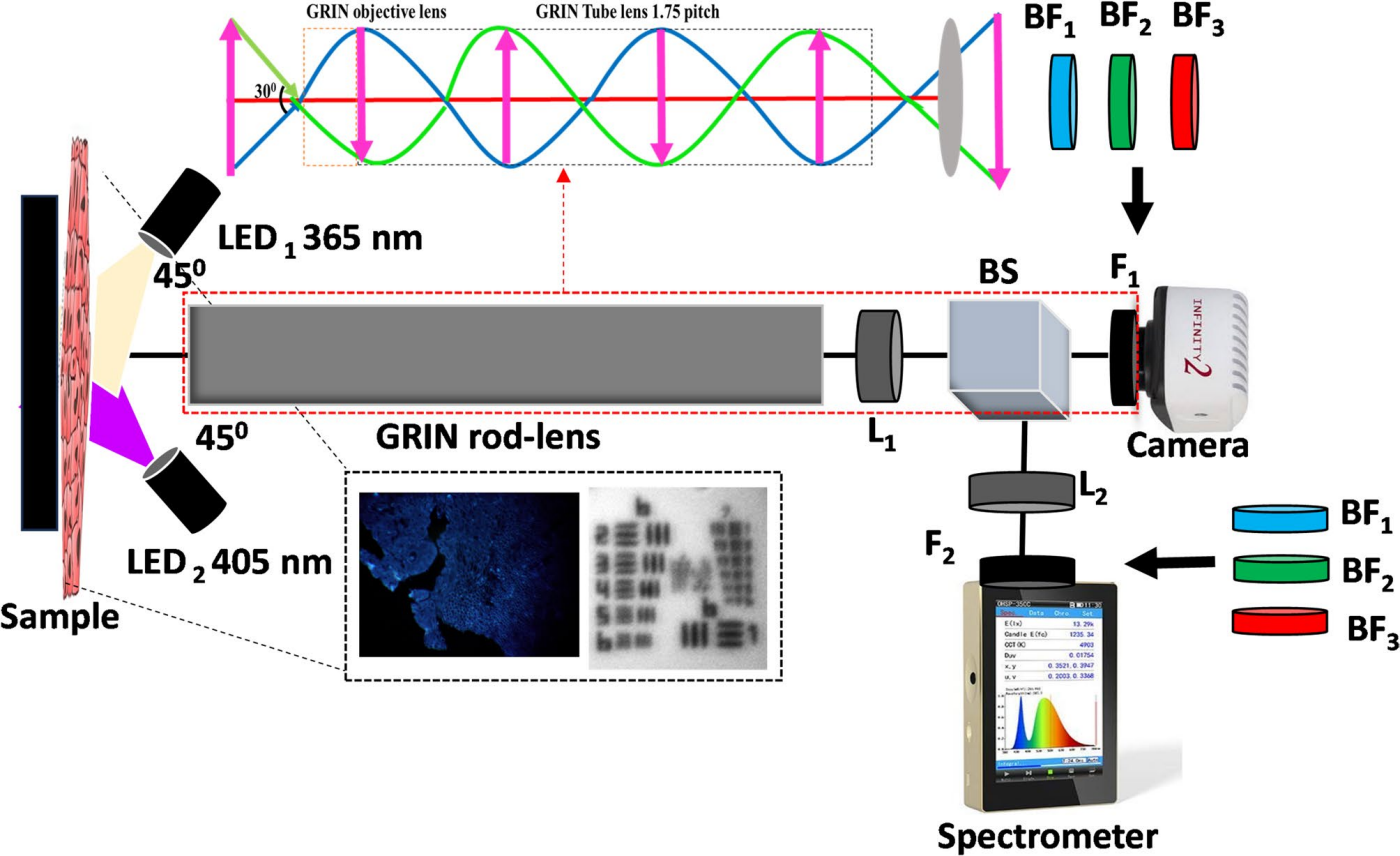
Experimental setup of the developed MSE for AF imaging and spectroscopy. The MSE contains two excitation LEDs of 365 and 405 nm with a GRIN rod-lens (GRIN, GT-ERLS-200-005-175-NC, diameter 2 mm, working distance 5 mm), two lenses (L_1_ and L_2_), each with 50 cm focal length, a 50–50 beam splitter (BS), long-pass filters (F_1_ and F_2_), band-pass filters (BF_1_, BF_2_, BF_3_) a spectrometer and a camera. Multispectral AF microscopy is done using 365 nm LEDs in the excitation, and three different band-pass filters, blue (450–490 nm), green (510–560 nm), and red (600–650 nm), are used in the emission to record AF images in each band. Simultaneous AF microscopy and AF spectroscopy are done using a 405 nm LED in excitation with long-pass filters, > 425 nm, in each arm of the MSE after BS, and AF images and spectra are recorded simultaneously. The inset shows an AF image of proteinuria tissue recorded with the setup, along with an image of a USAF resolution chart.

A gradient refractive index (GRIN, GT-ERLS-200-005-175-NC, diameter 2 mm, working distance 5 mm) lens-based MSE with oblique-back illumination was developed. The developed MSE is used for AF imaging and spectroscopy. Figure 1 indicates the experimental setup for the AF imaging and spectroscopy with the oblique illumination at 45 degrees with two light-emitting diodes (LEDs), 365 nm (< 5 mW) and 405 nm (< 5 mW). The AF images and spectra are recorded manually for these two channels. Firstly, the sample is excited with a 365 nm LED, and then the light gets scattered. Then, the GRIN rod lens collects the scattered signal, and a BS splits the signal into two arms, one for imaging and the other for spectroscopy. Lens L1 relays the signal at the imaging plane, where the camera captures the image after the filter F1 (long pass filter, > 425 nm). Lens L2 focuses the signal at the spectrometer, and after filter F2 (long pass filter, > 425 nm), the spectrometer records the signal simultaneously. In the second mode, three different bandpass filters (BFs) are used to probe different intrinsic fluorescent proteins. For this purpose, blue (450–490 nm), green (510–560 nm), and red (600–650 nm) filters were used. The same procedure as used for 365 nm excitation was utilized for 405 nm. The inset shows the USAF resolution chart, and the fifth element of the sixth group is resolved clearly. The MSE has a 4.92 μm spatial resolution, as shown in the inset of Fig. 1.

### Tissue preparation

The present study utilizes kidney samples from the lupus-prone Murphy Roths Large (MRL) strain MRL/MpJ-*Fas*^*lpr*^/J (MRL-lpr) obtained from Jackson Laboratories and conducted in accordance with the guidelines of the Norwegian Ethical and Welfare Board for Animal Research. The MRL-lpr strain develops systemic autoimmunity and immune complex glomerulonephritis resembling human LN, resulting in kidney inflammation and damage [9]. In the context of studying nephritis, ANA antibodies and specifically anti-dsDNA antibodies (ab) develop in the MRL-lpr strain from 12 weeks old (wo). At approximately 4 weeks, MRL-lpr mice are deemed young and free from disease. However, by 20 weeks, these mice produce a high amount of autoantibodies and protein in the urine, indicated by 4 + measured by Uristix (Bayer Diagnostics, Bridgend, United Kingdom). 0–1+ (< 1 g/liter) was regarded as physiological proteinuria, 2 + ≥ 1 g/liter to 3 g/liter, 3 + ≥ 3 g/liter to 20 g/liter, and 4 + ≥ 20 g/liter, where 3 + and 4 + were regarded as a proteinuric state. Anti-dsDNA antibody production was determined by an in-house ELISA^27^ OD450 over 0.2 was regarded as positive, and a cutoff of 40% of a positive control determined the anti-dsDNA antibody titer. Formalin-fixed kidney samples from young, antibody-positive, and proteinuric mice were obtained and prepared on standard microscopic slides with a thickness of 10 μm for AF spectroscopy and multispectral AF microscopy. Renal tissues from a total of 5 mice with proteinuria, 5 mice with positive anti-dsDNA titers (antibody-positive), and 6 young mice renal tissues were used in the study (Table 1). Three different regions of the tissues, including the cortex, medulla, and middle (between cortex and medulla), were analyzed. Table 1 shows the total sample points recorded by AF imaging and spectroscopy. The sample size used in the present study follows the Mead’s resource equation, with error degrees of freedom defined as the total number of animals subtracted with the three groups (young, antibody-positive and proteinuric). Recommended values of error degrees of freedom typically lie between 10 and 20 to balance statistical reliability with ethical consideration in animal experimentation. For 5 animals per group, the error degree is 12 and falls between the recommended values of Mead’s resource equation^28^. Though expanding the sample size will increase the statistical power and reduce the influence of biological variability and can be further validated.

**Table 1:** Description of the lupus-prone mice with indicated sample points.

S.*N*.	Cases	Strain	Age	Anti-dsDNA ab	Proteinuria	Number of mice/tissue sections	Total sample points
**1.**	Young	MRL-lpr	4–11 wo	Neg	Neg	6	63
**2.**	Antibody positive	MRL-lpr	20 wo	Titer > 1600	+ 1	5	72
**3.**	Proteinuric	MRL-lpr	20 wo	Titer > 3200	+ 4	5	70
	Total					16	205

### Image processing and statistical analyses

Captured images were processed by using MATLAB R2024b software to investigate AF information from the renal tissues. Firstly, we calculated the mean values from all samples of each case, shown in Table 1. For tissue from proteinuric mice, we calculated 5 different mean values in tissues from 5 different MRL-lpr mice for each AF microscopic image. After calculating these values, we averaged all of them and plotted the box plots showing intensity count variation on the y-axis with their respective standard deviation. For antibody-positive and young tissues, 5 and 6 different mean values are calculated according to the number of mice in each category, and the rest of the procedure is followed as for proteinuria tissues. The Analysis of variance (ANOVA) test was used to check the significance of statistical variance in the mean value of each proteinuric, antibody-positive, and young tissue case. After that, a Tukey’s Honestly Significant Difference (HSD) test was conducted on these tissues.

## Results and discussion

### Autofluorescence microscopy using 365 nm

**Fig. 2:**
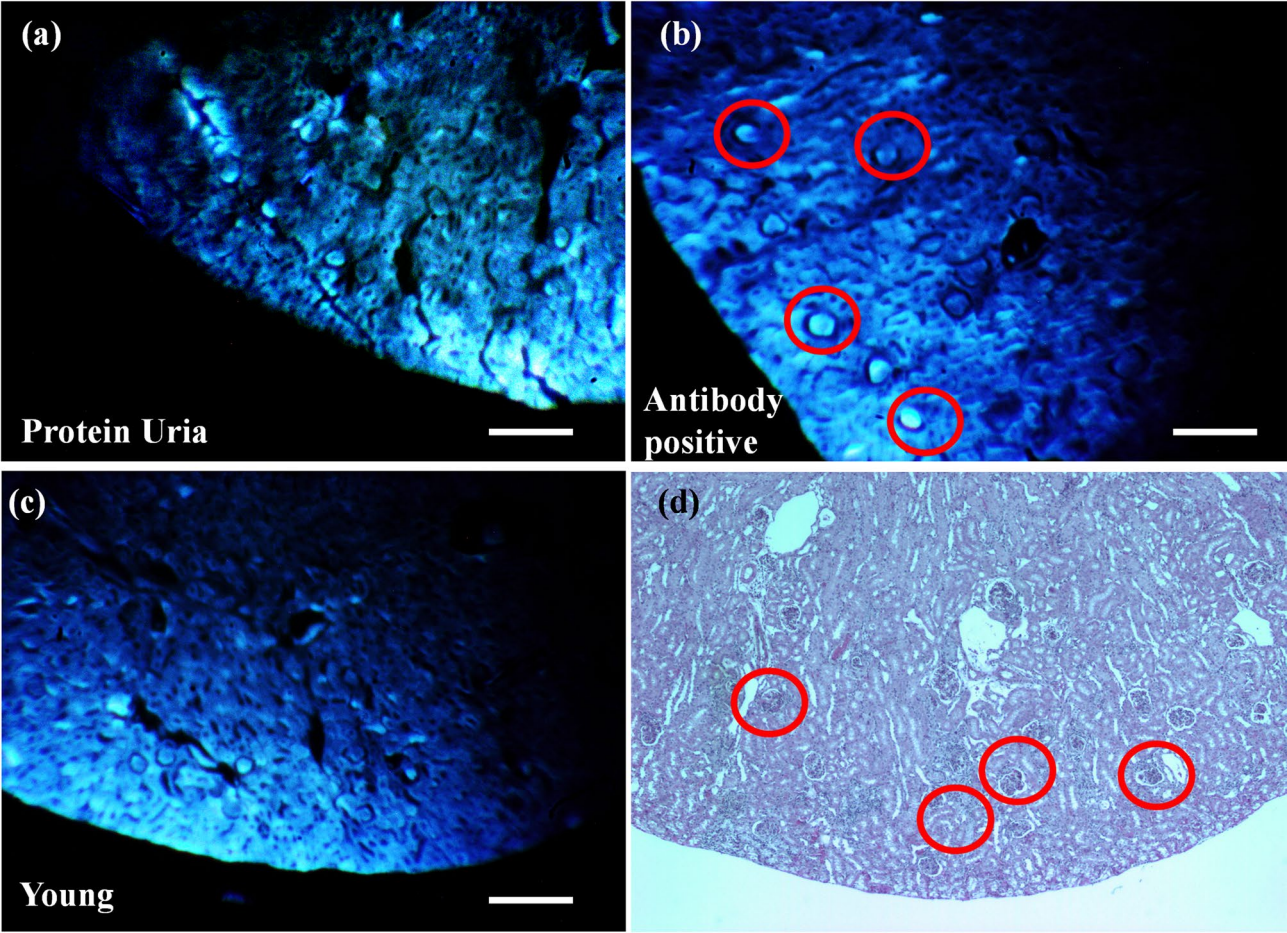
A comparison of the AF images of different renal tissue sections at 365 nm wavelength showed at identical experimental conditions (excitation power of 365 nm wavelength and exposure time). (**a**) AF image of kidney tissue from a proteinuric MRL-lpr mouse showing low AF intensity. (**b**) The AF image of kidney tissue from an antibody-positive MRL-lpr mouse shows the highest AF, with encircled glomeruli in red, showing high AF compared to the background. (**c**) AF image of kidney tissue from a young MRL-lpr mouse showing high AF intensity at the kidney capsule. (**d**) The histopathological image of an H&E-stained kidney section from an antibody-positive MRL-lpr mouse was taken at 4X magnification, and some of the glomeruli are depicted in red circles. Scale bar = 200 μm.

Autofluorescence microscopy was first performed using the 365 nm excitation. Figure 2a–c shows the AF images of renal tissues from proteinuric, antibody-positive, and young mice filtered by the long pass filter 425 nm. The AF intensity of the tissue from proteinuric and antibody-positive mice was higher compared to the young (Fig. 2). Both antibody-positive and proteinuric mice have been reported to have deposition of anti-dsDNA ab in glomeruli and in tubulointerstitial areas^29^. It is, therefore, difficult to link the production of ANAs to the high amount of AF observed in antibody-positive tissue. However, in renal tissues, the proximal tubules are responsible for reabsorbing most of the filtered water, sodium, glucose, amino acids, and other valuable organic substances from the primary filtrate. In those proximal tubules, a dense concentration of mitochondria exists^20,30^. These mitochondria play a crucial role by generating adenosine triphosphate, the energy currency essential for facilitating extensive solute transport in this particular segment of the nephron^31^. Mitochondria are recognized for emitting AF signals attributed to NADH and FAD. When in its reduced state NADH, NAD emits fluorescence in the blue range (420–480 nm), serving as a vital cofactor in the citric acid cycle, beta-oxidation of fatty acids, and as the substrate for complex I in the respiratory chain^31^. NADH plays a crucial role in macromolecule synthesis and antioxidant defense maintenance, sharing identical excitation/emission wavelengths with NADH. During the development of end-stage kidney disease with subsequent proteinuria, a reduction in tubular reabsorption was reported^32^. The observed reduction in the AF signal may be explained by a reduction in both NADH and NAD+. Oxidative stress and inflammation cause enzymes like PARPs and CD38 to consume more NAD⁺, which lowers NAD⁺ levels as the disease progresses^33^. Furthermore, the salvage and de novo synthesis pathways are disrupted by poor NAD⁺ production in proximal tubular epithelial cells, which further lowers availability^33,34^. Mitochondrial malfunction worsens this depletion, resulting in energy shortages and accelerating disease progression^20,30^. The availability of NAD⁺ precursors, including nicotinamide and nicotinamide riboside, is also restricted by nutritional deficits and metabolic abnormalities. Cellular malfunction, fibrosis, and accelerated renal aging are all influenced by this NAD⁺ decrease^35^.

Flavins like FAD and flavin mononucleotide are significant redox cofactors emitting in the green range (520~560 nm) when oxidized, with their fluorescence characteristics reliant on the specific flavoproteins in which they are situated^36^. In addition to NADH and FAD, collagen also shows autofluorescence after exciting with 365 nm wavelength^37^. The cortex in the kidneys mainly consists of glomeruli and proximal and distal convoluted tubules, and contains collagen and shows increased AF compared to the background tissues^38^. This increased AF in glomeruli due to the collagen and FAD correlates with the experimental observation of each case of MRL strain (young, antibody-positive, and proteinuric) shown in AF microscopic image Fig. 2b. The brightfield image was recorded using H&E-stained tissue and is shown in Fig. 2d. Glomeruli are tiny networks of blood vessels, and their main function is to filter out the waste and extra fluids from the blood into the urinary space^39^.

Formalin fixation can affect tissue autofluorescence by altering fluorescence intensity and spectral characteristics of endogenous fluorophores. Previous studies have shown that intrinsic metabolic cofactors remain detectable in formalin-fixed and FFPE tissue sections, enabling autofluorescence-based analyses under controlled conditions^40^. However, fixation can complicate direct attribution of individual fluorophores, particularly within the NADH and FAD emission ranges, and these effects depend on tissue type, fixation duration, and chemical protocol. In this study, all renal tissue sections were processed using an identical fixation method and exposure time; therefore, although fixation may affect absolute autofluorescence properties, its influence is expected to be uniform across samples, preserving the validity of relative tissue comparisons^40,41^.

### Autofluorescence microscopy for different renal regions

**Fig. 3:**
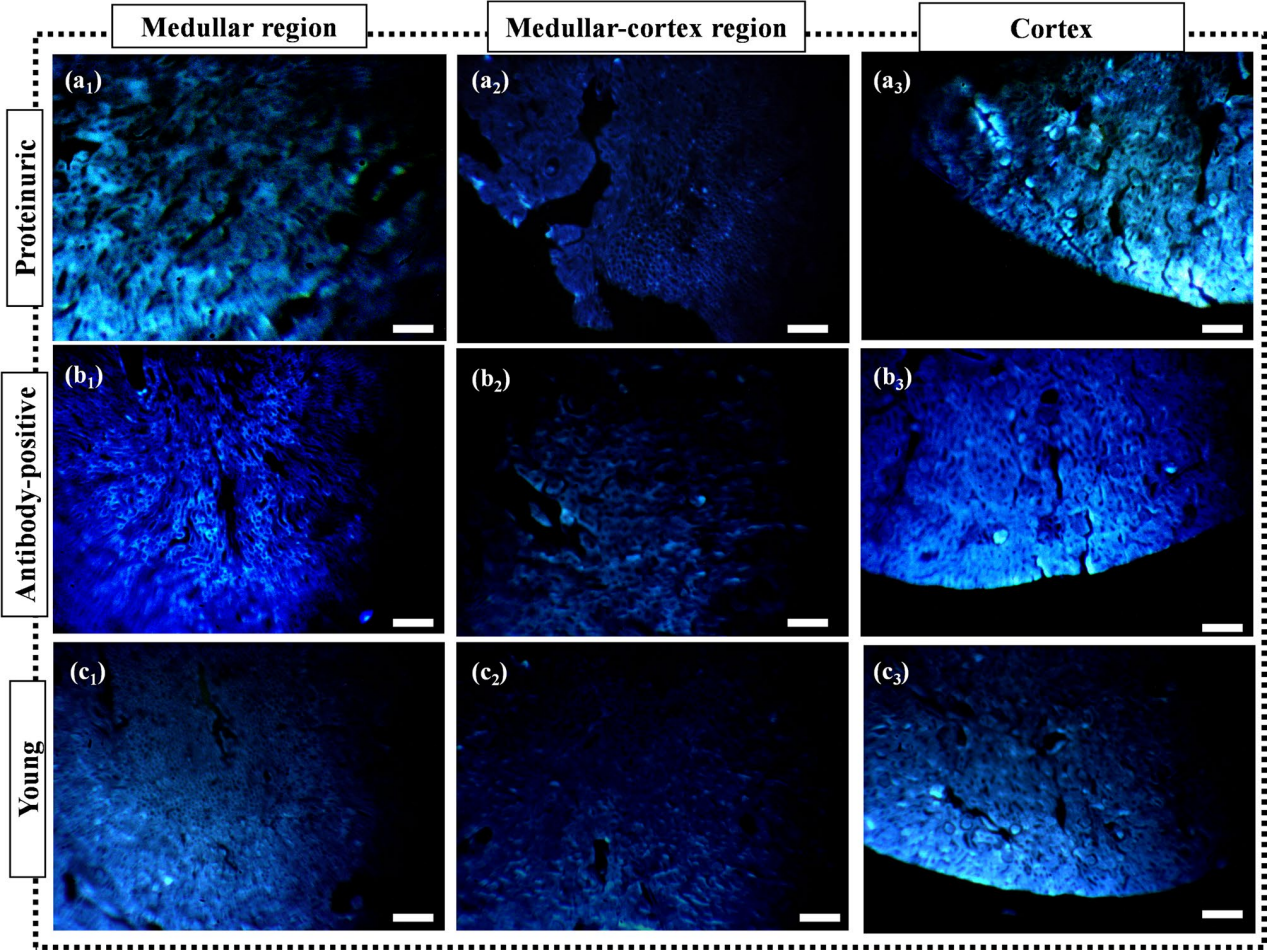
Autofluorescence (AF) images of kidney tissue from MRL-lpr mice following excitation at 365 nm. (**a1**–**c1**) AF images of the medulla region from proteinuric (**a1**), antibody-positive (**b1**), and young (**c1**) MRL-lpr mice. (**a2**–**c2**) AF images of the intermediate region between medulla and cortex from proteinuric (**a2**), antibody-positive (**b2**), and young (**c2**) MRL-lpr mice. (**a3**–**c3**) AF images of the cortex region from proteinuric (**a3**), antibody-positive (**b3**), and young (**c3**) MRL-lpr mice. The images show AF variation in different tissue regions with each disease category. scalebar=200 μm.

**Fig. 4:**
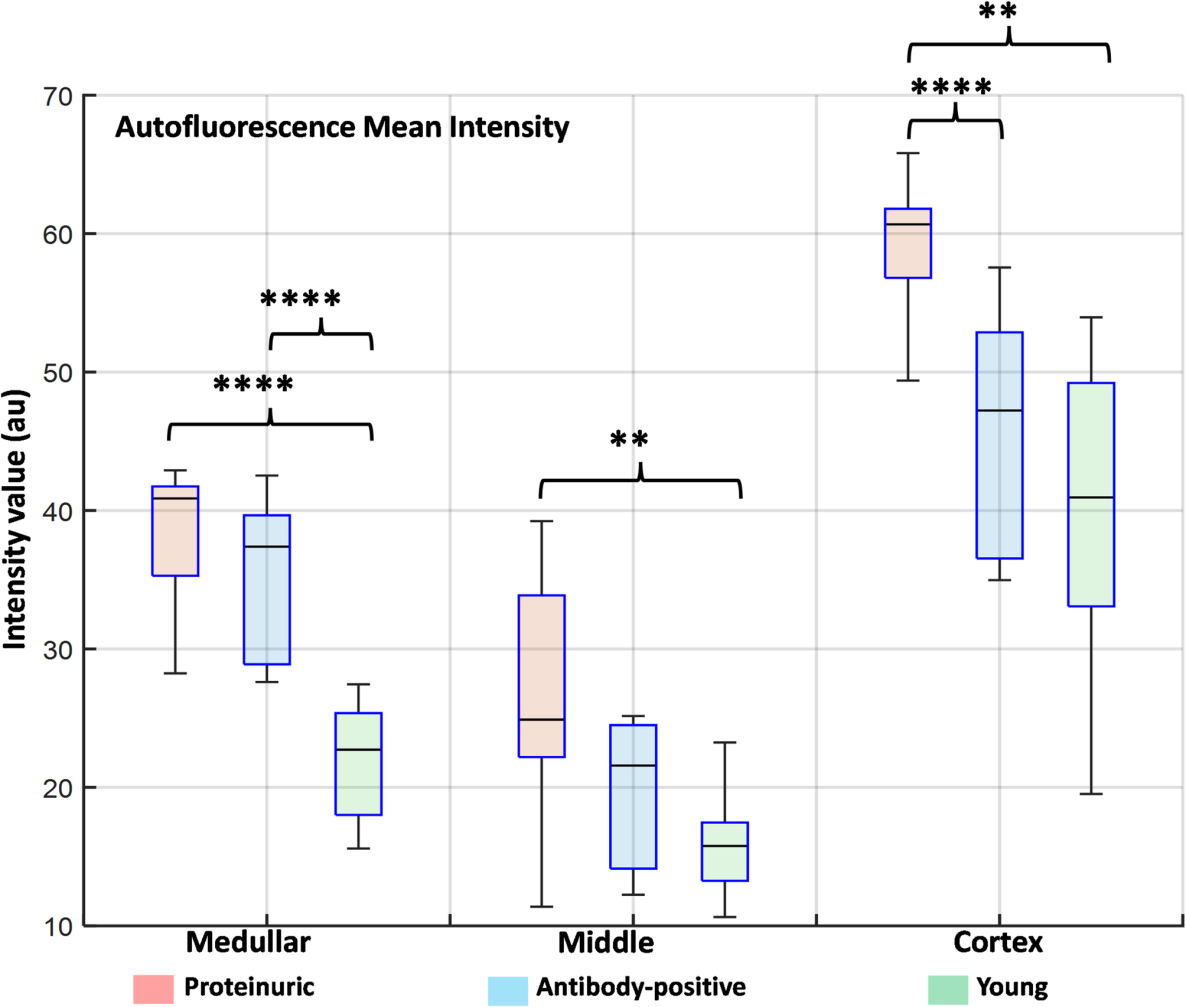
AF intensity variations in the Medulla, Middle, and Cortex regions across Young, Antibody-positive, and Proteinuric. Boxplots show AF intensity distributions by region and tissue type. Significant differences across comparisons are marked by asterisks, with the cortex region consistently showing the highest intensities across all groups. Proteinuric tissue exhibits significantly elevated intensity in the medulla and cortex compared to other groups. The most prominent differences were observed in the cortex between Proteinuric and Young (*p* < 0.0001) and Proteinuric vs. Antibody-positive (*p* = 0.001), as well as in the medulla between Proteinuric and Young (*p* < 0.0001) and antibody-positive and young.

To further examine the AF property of renal tissues obtained from proteinuric, antibody-positive, and young MRL-lpr mice, a total of three different regions, medulla, medulla-cortex, and cortex, were recorded using AF microscopy (Fig. 3). Increased AF was observed in all tissue regions analyzed from the proteinuric mice compared to the antibody-positive and young MRL-lpr mice (Fig. 3). The medullary region is mainly responsible for the regulation of urine concentration^42^. The kidney samples analyzed displayed significant AF, especially within the proximal tubule, and this may be attributed to numerous natural fluorescent compounds. The medulla region shows more AF in proteinuric and antibody-positive compared to the young, as seen in Fig. 4. NADH serves as the starting material for complex I within the respiratory chain and participates in various cellular redox reactions^30^. NADH exhibits fluorescence in its reduced form, unlike its oxidized state, NAD+, which is a valuable indicator of the redox state within mitochondria^30,35,43^. Figure 3a_1_ and b_1_ show increased AF compared to (c_1_). The reason behind it can be due to the presence of NADH and collagen, and their concentration changes during the disease progression^35,38^.

Most of the AF signals are due to the changes in metabolites at the intracellular level and can be associated with mitochondria, and show a higher probability that AF comes from it ^20,30^. Figure 3a_2_–c_2_ shows the tissue region between the medulla and cortex. Increased AF contrast was observed in the medulla of proteinuric mice compared to the tissue from antibody-positive and young MRL-lpr mice (Fig. 3a_2_). The cortex regions showed increased AF at the edge of the tissue sections (Fig. 3a_3_–c_3_). This could be because the cortex is surrounded by fatty acid layers within the renal capsule^44^. In the renal capsule, the metabolic activity due to the redoxation of NADH is varied, thus resulting in AF contrast in the different types of cases. The cortex of the kidneys is mainly made up of glomeruli and proximal and distal tubules. In LN, the glomeruli are primarily affected by the deposition of immune complexes, leading to increased inflammation and the progression of proteinuric disease^45^. The renal cortex, particularly the glomeruli, shown in Fig. 2b and d, with red circles in charge of removing waste and extra fluid from the circulation to create urine. These regions are usually impacted by inflammation. Proteins ordinarily kept in the blood can enter the urine due to damage to the glomeruli, leading to proteinuria^45^. In advanced LN, inflammation and scarring may spread to the renal medulla, disrupting the kidneys’ ability to regulate electrolytes and concentrate urine, leading to fluid retention and electrolyte imbalances^42,45,46^.

#### Statistical analysis

A two-way ordinary ANOVA was performed to assess the effects of group (row factor) and tissue region (column factor), as well as their interaction, on the measured variable (AF Intensity). The analysis in Table S1 in supplementary shows that all main effects and their interaction were statistically significant. The interaction between group and tissue region accounted for 2.61% of the total variation and was significant (F(4, 90) = 2.95, *p* = 0.0243), indicating that the effect of one factor varied depending on the level of the other. The group factor contributed 18.29% of the variation (F(2, 90) = 41.35, *p* < 0.0001), while the tissue region factor contributed the largest portion of variation at 59.24% (F(2, 90) = 133.88, *p* < 0.0001). The analysis was based on 99 observations across three groups and three tissue regions.

To assess the simple effects within each tissue region, post hoc comparisons using Tukey’s HSD test were conducted. Table S2 in supplementary shows the detailed Tuckey’s test parameters extracted from the AF signal of proteinuric, antibody-positive, and young tissues. In the cortex, significant differences were observed between antibody-positive and proteinuric groups (mean difference = -13.54, *p* < 0.001), and between proteinuric and young groups (mean difference = 19.59, *p* < 0.0001), whereas the comparison between antibody-positive and young groups was not significant (*p* = 0.24). In the medulla, antibody-positive mice had significantly higher values than young mice (mean difference = 13.25, *p* < 0.0001), and proteinuric mice also differed significantly from young mice (mean difference = 15.92, *p* < 0.0001); however, the difference between antibody-positive and proteinuric groups was not significant (*p* = 0.41). In the middle region, only the comparison between proteinuric and young groups was statistically significant (mean difference = 10.38, *p* < 0.001), while the other comparisons were not. These findings suggest that group differences were most pronounced in the cortex and medulla regions, particularly between proteinuric and young tissues.

Figure 4 shows the ANOVA and post hoc comparison results, highlighting AF intensity counts in the different renal regions in tissue from proteinuric, antibody-positive, and young MRL-lpr mice. In Fig. 4, we can see that the overall highest AF value was observed for the cortex region for each representative tissue, probably due to the presence of glomeruli, as seen in Fig. 4. The kidney’s cortex, which includes collagen and exhibits higher AF than the surrounding tissues, is mostly made up of glomeruli and proximal and distal convoluted tubules^38^. For each renal region, proteinuric mice showed the highest AF, followed by tissues from antibody-positive and young MRL-lpr mice. The mature renal medulla consists of the medullary collecting ducts, loops of Henle, and vasa recta (straight capillaries), as well as the interstitium, which contains lipid-laden interstitial cells, lymphocyte-like cells, and pericytes^31^. As proteinuria increases in lupus nephritis, the lipid metabolism is disturbed. This leads to an elevation in serum triglycerides, very low-density lipoproteins (VLDL), and intermediate-density lipoproteins (IDL)^47^. Due to the increment of these lipoproteins, the autofluorescence rises in all three regions: medulla, cortex, and middle in the case of tissues from proteinuric and antibody-positive MRL-lpr mice, and the least for the renal tissues from young MRL-lpr mice^37^. The cortex showed the highest AF intensity difference between proteinuric and both young and antibody-positive tissues (*p* < 0.0001 and *p* = 0.001, respectively). The medulla displayed similarly high differences between proteinuric and young tissues (*p* < 0.0001). In contrast, the middle region showed a more moderate difference only between proteinuric and young mice (*p* = 0.001), indicating that the cortex and medulla contribute most prominently to AF-based distinction among disease stages. Together, these analyses show that proteinuria in lupus-prone mice correlates with significant and region-specific increment in renal AF, particularly in the cortex and medulla.

In interpreting these tissue region difference, it is important to consider potential non-pathological optical contributors. Further, the AF images were not corrected for vascular density and thus could lead to difference in hemoglobin absorption and scattering sometimes. The present AF measurements were performed on ex-vivo renal sections under similar acquisition conditions therefore minimizing the dynamic vascular influences as perfusion/oxygenation. Therefore, while non-pathological factors like optical absorption or scattering cannot be completely excluded, they are unlikely to solely account for the observed differences in AF for the tissue regions.

### Multispectral autofluorescence microscopy

**Fig. 5:**
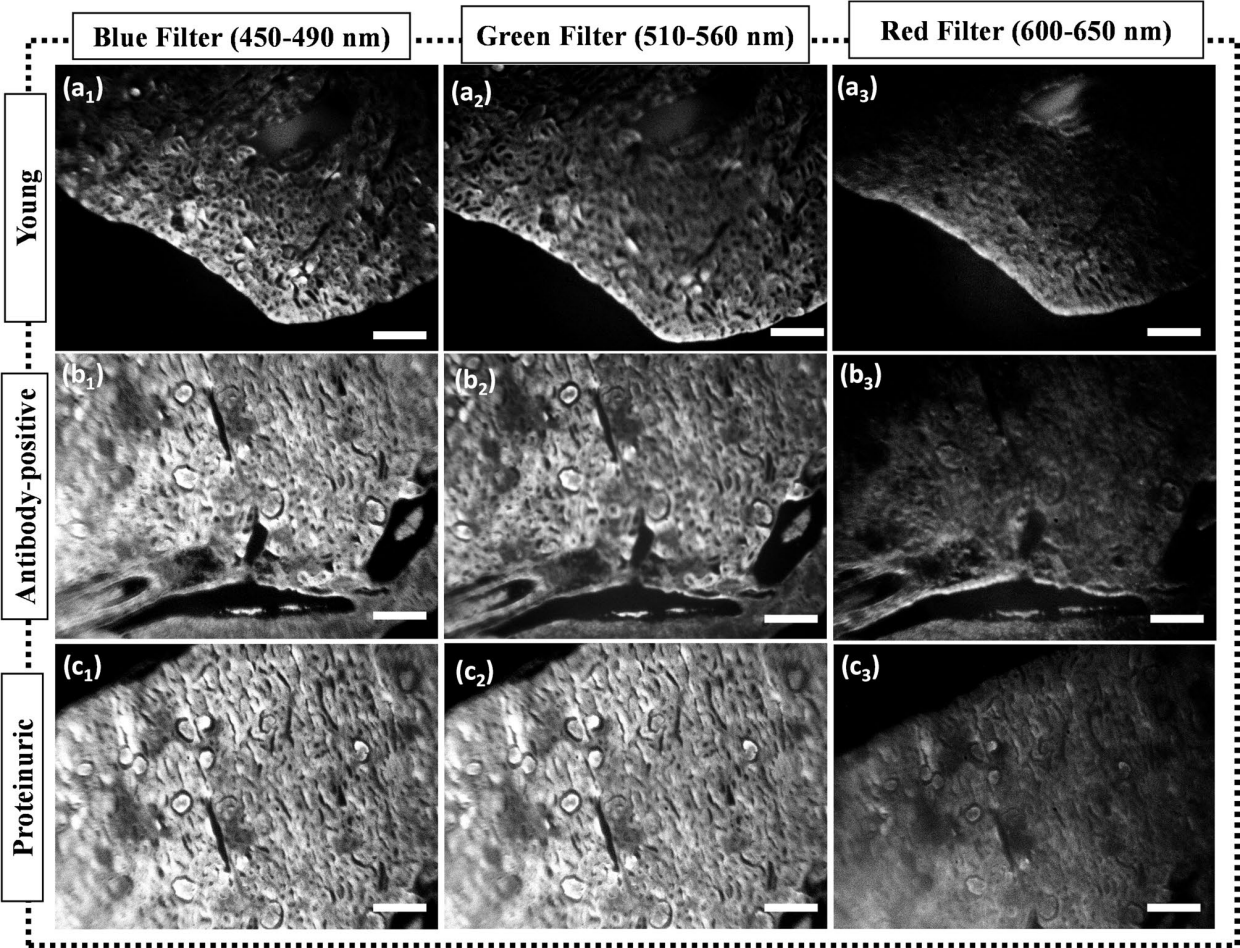
Comparison of autofluorescence (AF) images of kidney tissue from MRL-lpr mice acquired in three emission bands (450–490 nm, 510–560 nm, and 600–650 nm) following 405 nm excitation. (**a1**–**a3**) AF images from a proteinuric MRL-lpr mouse using blue, green, and red emission filters, respectively. (**b1**–**b3**) AF images from an antibody-positive MRL-lpr mouse using the same filters. (**c1**–**c3**) AF images from a young MRL-lpr mouse using the same filters. Images show spectral differences in AF signal distribution and intensity corresponding to disease progression. Scale bar = 200 μm.

We used the cortex region of the renal tissues to explore and investigate the AF information using different spectral bands. The main reason to look into the cortex is that nephrons are present and affected during the disease’s progression. The multispectral autofluorescence microscopy of these tissues was done using 365 nm excitation. Three different optical bandpass filters were used to capture multispectral AF images from the renal tissues. Figure 5 shows AF images of proteinuric, antibody-positive, and young tissues using three emission filters to probe different intrinsic fluorophores. Figure 5a_1_–a_3_ shows the AF images of the proteinuric tissues in blue (450–490 nm), green (510–560 nm), and red (600–650 nm) spectral ranges, respectively, and shows the difference and variation of AF intensity in all ranges. Proteinuric kidneys had more intensity in the blue region, probably due to the presence of NADH, compared to the tissues from antibody-positive and young MRL-lpr mice. Figure 5b_1_–b_3_ shows AF images of tissues from antibody-positive mice in blue (450–490 nm), green (510–560 nm), and red (600–650 nm) spectral ranges, respectively. The AF in the green band for the antibody-positive mice was greater compared to the other bands. Similarly, Fig. 5c_1_–c_3_ shows the AF images of young tissues in blue (450–490 nm), green (510–560 nm), and red (600–650 nm) wavelength ranges, respectively. To enhance image contrast for better visualization, all figures were uniformly adjusted to a constant value using ImageJ software.

Figure 5 shows that the highest AF intensity was recorded using the green filter on tissues from proteinuric mice in blue and green filter followed by tissue from the antibody-positive mice, while the tissue from the young MRL-lpr mice, where no disease was present, showed less AF. The elevation in AF intensity correlated with increased levels of ANA antibodies in proteinuric and antibody-positive cases. This observation is supported by earlier results shown in Fig. 3, where proteinuric mice had the highest AF in the cortex compared to the other mice groups. Figure 5a_1_–c_1_ gives the AF in the blue region, indicating the presence of NADH in these renal tissues^1–3,12,48^. The AF intensity of young tissue in the blue spectral band, as shown in Fig. 5a1, seemed higher compared to green and red spectral bands, because when the disease in renal tissues like proteinuria, there is a decrease in NAD+ levels that probably results from a combination of reduced NAD+ biosynthesis and increased NAD+ consumption. The increased levels of NAD⁺ are probably the cause of the enhanced AF intensity seen in young tissue within the blue spectral band (Fig. 5, a1)^49^. Furthermore, in the spectral band of 510–560 nm (green filter), the AF intensity was higher for each case of renal tissues, giving an idea about the redoxation of NADH-FAD crosslinks presence. The higher AF intensity in the green frame reflects elevated FAD and flavoproteins (FP) levels due to autoantibody deposition, heightened immune cell infiltration, and altered uptake of biological materials by the proximal tubule. The renal capsule comprises a stratum of stromal cells enveloped by a layer of connective tissue, exerting significant influence throughout kidney ontogeny and adult renal equilibrium^50^.

The spectral band 600–650 nm (red filter), as in Fig. 5b_3_ and c_3_, showed the AF at the edges of young and antibody-positive tissues and the renal capsules. This indicates higher porphyrin uptake in young and antibody-positive tissues compared to proteinuric, which are primary components of hemoglobin^51^. This observation in Fig. 5a_3_ and b_3_ indicates increased levels of Hb in these tissues compared to proteinuric, contributing to the distinctive AF intensity patterns observed in these renal samples. During the disease progression (young to antibody), the porphyrins are aggregated in the renal tissues^52^. Porphyrins exhibit limited filtration by dialysis membranes, likely attributable to their high affinity for plasma albumin and hemopexin binding^53^.

#### Statistical analysis

A two-way ANOVA was performed to assess the effects of group and tissue region, and their interaction, on the dependent variable (α = 0.05). The analysis revealed a significant interaction between group and tissue region (F(4, 228) = 5.85, *p* = 0.0001), accounting for 5.14% of the total variance. This suggests that the effect of one factor depends on the level of the other. Both main effects were also highly significant for Group: F(2, 228) = 52.02, *p* < 0.0001 (22.84% of variation) and Tissue region: F(2, 228) = 49.99, *p* < 0.0001 (21.95% of variation). The residual error accounted for the remaining 49.9% of the variance, indicating substantial within-group variability. These results highlight strong and independent effects of both factors, as well as their interaction.

Tukey’s HSD test identified significant pairwise differences in all three tissue regions. In the blue region, the young group showed significantly lower values than both antibody-positive (MeanDiff = − 20.01, *p* < 0.0001) and proteinuric animals (MeanDiff = − 22.31, *p* < 0.0001), while the difference between antibody-positive and proteinuric was not significant (*p* = 0.59). In the green region, young animals again showed significantly lower values than both antibody-positive (MeanDiff = − 8.41, *p* = 0.0165) and proteinuric (MeanDiff = − 11.28, *p* = 0.0009), with no significant difference between the two disease groups (*p* = 0.66). In the red region, a significant difference was observed between antibody-positive and young animals (MeanDiff = − 12.54, *p* < 0.0001). Comparisons involving proteinuric animals (vs. antibody-positive and young) were marginally non-significant (*p* = 0.0612 and *p* = 0.0627, respectively). Overall, young animals consistently exhibited lower values, while antibody-positive and proteinuric groups did not differ significantly, suggesting a shared pathological phenotype. Table S3 and S4 in supplementary shows detailed analysis of two-way ANOVA and Tukey’s HSD test.

**Fig. 6:**
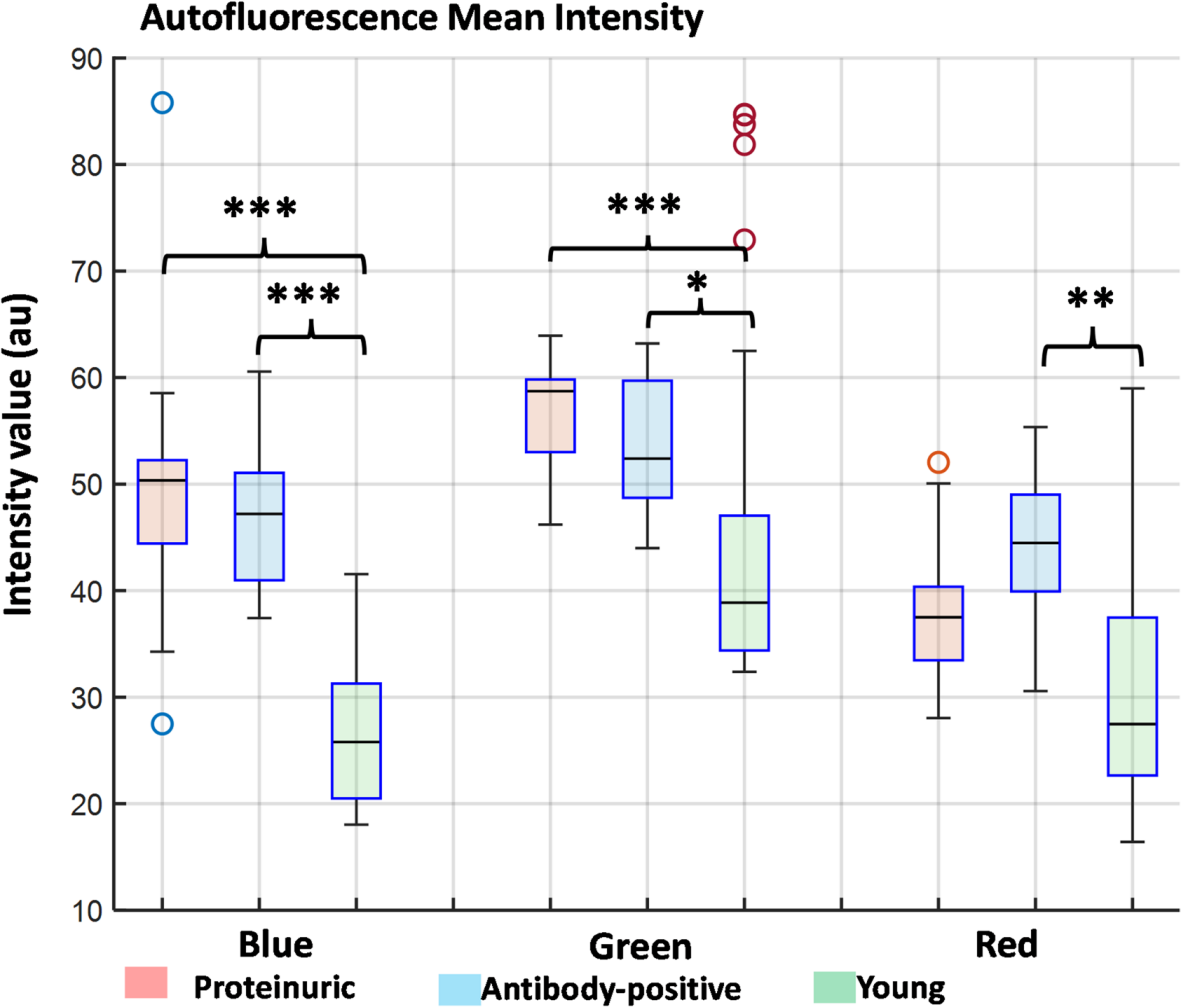
AF intensity differences across blue, green, and red filters in kidney tissue from proteinuric, antibody-positive, and young MRL-lpr mice, based on two-way ANOVA and Tukey’s HSD post hoc tests. (a) Boxplots indicates that AF intensity increases progressively from young to antibody-positive and is highest in proteinuric mice across blue and green filters. Statistically significant differences are observed between proteinuric and young tissues under blue (*p* < 0.001), and green (*p* < 0.001), indicating wavelength-dependent changes in AF associated with disease severity. The green filter also shows significant difference between antibody-positive and young mice (*p* < 0.01). Red filter only shows significance between antibody-positive and young tissues. The strongest AF contrast was observed between antibody-young and proteinuric - young tissues under the blue filter (*p* < 0.0001), followed by proteinuric-young in green and antibody-young in red filters, confirming that spectral shifts in autofluorescence are sensitive to disease progression in lupus-prone kidneys.

Figure 6 shows the Tukey’s HSD post hoc test results following ANOVA to assess pairwise differences between the blue, red, and green filters in tissues from proteinuric, antibody-positive, and young MRL-lpr mice. Each colored box represents the distribution of data within a specific group (proteinuric, antibody-positive, and young), with median, interquartile range, and potential outliers depicted. The groups are further differentiated using color coding, red, blue, and green, corresponding to three disease categories, proteinuric, antibody-positive, and young tissues, respectively, as shown in the legend. The horizontal brackets connecting pairs of box plots indicate comparisons where Tukey’s HSD test was applied. Asterisks above the brackets denote the statistical significance levels of the differences observed between the paired groups. **** indicates *p* < 0.00001, *** indicates *p* < 0.0001 and ** indicates *p* < 0.001. These annotations suggest that these inter-group comparisons yielded statistically significant differences in mean values, reflecting real, non-random variations across the experimental conditions. Within the blue and green filter, proteinuric and antibody positive tissues are highly significant with young tissues, suggesting that there is spectral dependence of disease progression in MRL-lpr mice in blue and green region. In Red filter, only antibody and young tissue show statistical significance.

### Simultaneous autofluorescence spectroscopy and microscopy

**Fig. 7:**
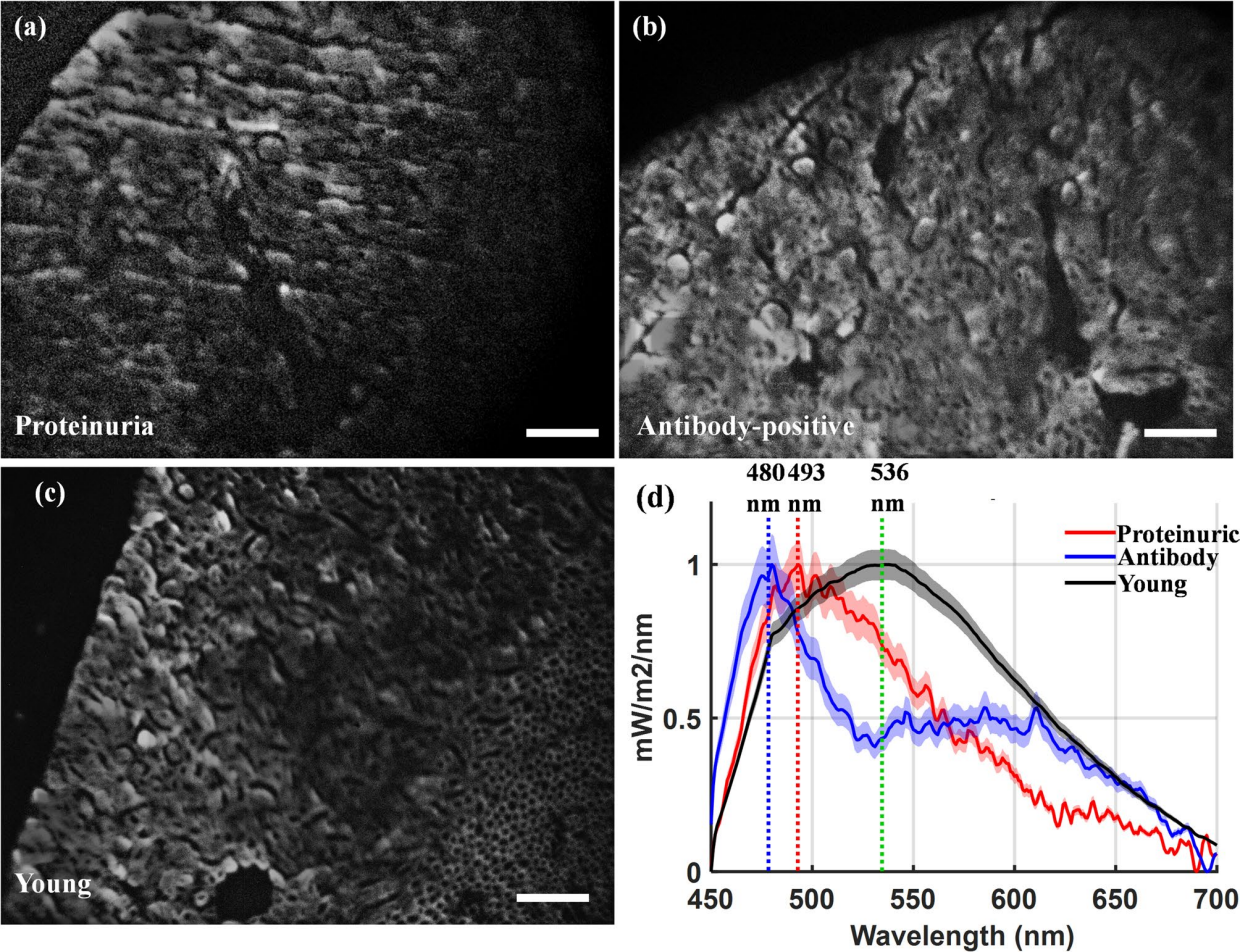
Simultaneous AF microscopic images and spectra of renal tissues were excited with 405 nm and emitted using a long pass filter at 425 nm. (**a**) AF image of renal tissue from a proteinuric MRL-lpr mouse showing the cortex region. (**b**) AF image of tissue from an antibody-positive mouse. (**c**) AF image of tissue from a young mouse. (**d**) The average spectra of in total 205 tissue spectra were taken from proteinuric, antibody-positive, and young MRL-lpr mice. Spectra show peak maximums at 493 nm for tissues from proteinuric mice, indicating emission of NADH. For antibody-positive mice, the peak maximum of the spectra was observed at 480 nm for NADH, and a new peak generation of around 616 nm may be responsible for AF from lipoprotein. The peak maximum for tissues from young mice was around 536 nm, which may be due to the NADH-FAD correlation. Scalebar=200 μm.

The simultaneous AF spectroscopy and microscopy were done by using the 405 nm wavelength. We used 405 nm as an excitation source to probe the porphyrins in the tissue region. We used a 425 nm long-pass filter in emission to record AF spectra and images. Figure 7 exhibits the simultaneous AF imaging and spectra of renal tissues. A total of 205 spectra were recorded for these three disease conditions out of a total of 16 cases. Figure 7a–c shows representative AF images of renal tissue from proteinuric, antibody-positive, and young MRL-lpr mice. AF intensity was highest for the antibody-positive mice, followed by kidney tissues from proteinuric and young mice. The peak maximum wavelengths in the AF spectra of tissues from proteinuric, antibody-positive, and young mice were measured at ~ 493 nm, ~ 480 nm, and ~ 536 nm, respectively, corresponding to FAD expression^1,2^, as shown in Fig. 7d. Figure 7d shows the average spectra from 70, 72, and 63 sample points from tissues from proteinuric, antibody-positive, and young mice. Notably, these peak shifts may indicate metabolic alterations during disease progression. Additionally, in the case of antibody-positive kidney samples, a new peak emerges around ~ 616 nm, indicative of lipofuscins^40,41,54^. This distinct peak may correspond to specific metabolic changes observed in tissue from antibody-positive animals. The AF spectroscopic analysis demonstrates the potential for diagnostic differentiation among disease progression, as evident from Fig. 7d. Peak shift in FAD in Fig. 7d may arise due to changes in the concentration of the fluorophores present in the tissue^1^. In the case of tissues from antibody-positive MRL-lpr mice, the AF spectral peak was observed around ~ 480 nm, indicating a blue shift compared to tissues from proteinuric mice (~ 493 nm) and young mice (~ 536 nm).

**Fig. 8:**
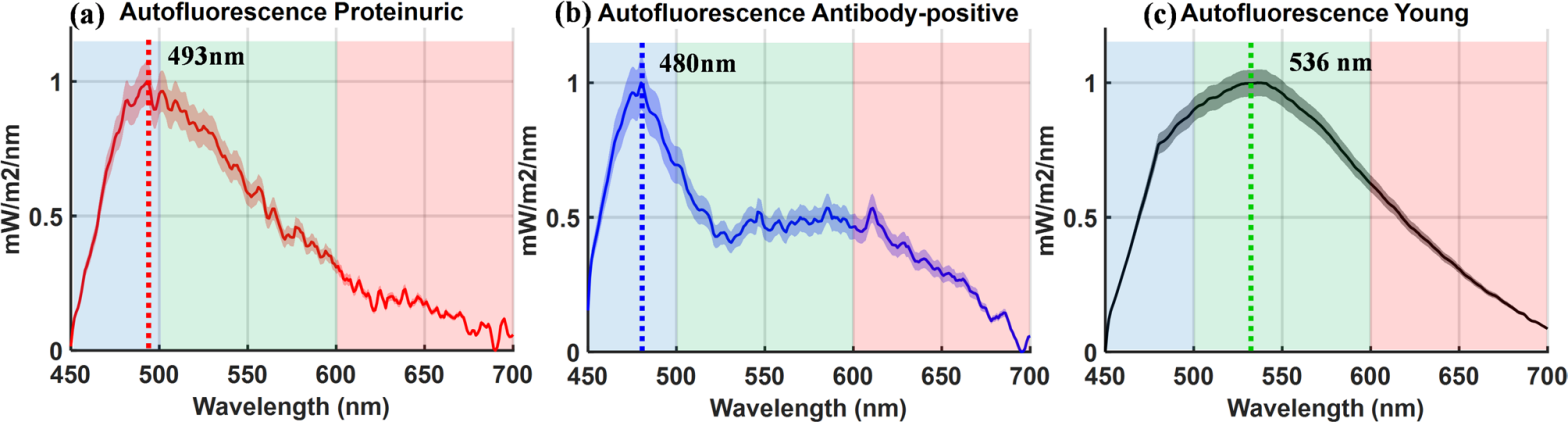
Averaged autofluorescence spectra of renal tissues from Proteinuric, antibody-positive, and young MRL-lpr mice. (**a**) Autofluorescence spectra of tissues from proteinuric mice. The maximum peak for NADH-FAD was ~ 493 nm. The blue band in (**a**) shows the presence of NADH-FAD crosslinks from 450 ~ 524 nm. The red band in the figure is responsible for porphyrins ranging from 621 ~ 700 nm. (**b**) Autofluorescence spectra of tissue from antibody-positive mice. The blue region from 450 ~ 520 nm is responsible for NADH-FAD, the green region from 531 ~ 600 nm is responsible for flavoproteins, and the red region from 622 ~ 700 nm is responsible for porphyrins in the tissue from antibody-positive MRL-lpr mice. (**c**) Autofluorescence spectra of tissue from young mice. The green band for the tissues from young mice was from 480 ~ 560 nm.

Figure 8 shows the individual spectra of proteinuric, antibody-positive, and young tissues of Fig. 5d. Figure 8a shows the tissue’s AF from proteinuric mice, particularly in blue (NADH-FAD crosslinks) from 450 ~ 524 nm. This spectral region is mainly for detecting the NADH-FAD crosslinks and sometimes collagen as well. Figure 8b shows the AF spectra for tissues from antibody-positive mice with a peak maximum of around ~ 480 nm, which was shifted compared to the peak observed for proteinuric mice and may be due to the difference in fluorophore concentration of NADH-FAD, lipofuscin and other fluorophores. Figure 6c shows the AF spectra of tissues from young mice with a peak maximum of ~ 536 nm, mainly from FAD excitations, but the effect of other fluorophores like NADH and lipofuscins cannot be neglected. The FWHM for young tissue is slightly higher compared to other two likely due to the change of fluorophore concentration in the tissue microenvironment and can be confirmed with the help of immunohistochemistry or biochemical detection. The additional AF spectra of the tissues can be found in Supplementary Fig. S3.

Here, we show that multispectral AF microscopy of the renal tissues taken from mice at different disease stages has different AF information for each case in different renal regions. The simultaneous spectra shows maximum peak shift for all three stages and peak shifts may mainly be due to the change in concentration of endogenous fluorophores^1,2^. The antibody-positive tissues showing a bump in the spectra starting around ~ 530 nm. The possible reason for change is increasing lysosomal activity and oxidative stress in the tissue^37,55^. Table 2 is showing the maximum peak wavelength and FWHM for all tissue regions.

**Table 2:** Peak wavelength, FWHM and AUC of the AF spectra.

S.N.	Tissue type	Peak wavelength (~ nm)	FWHM (~ nm)
1.	Young	493	100
2.	Antibody-Positive	480	155
3.	Proteinuric	536	148

These preliminary spectral differences highlight the potential of autofluorescence analysis for distinguishing stages of renal involvement. While the current findings are based on limited sample size, expanding such studies across larger cohorts could enable more detailed characterization of lupus nephritis progression and support the development of AF based diagnostic approaches.

The limitation of present study lies in the observed AF which represents the total emission arising from multiple endogenous fluorophores from tissue microenvironment including NADH, FAD, lipofuscin, collagen, porphyrins, without doing fluorophore specific biochemical validation. Thus, the molecular information from fluorophores is inferential rather than definitive. Knowing the intrinsic endogenous fluorophores AF spectral overlap, the observed AF is likely giving an idea about cumulative alterations in metabolic and structural constituents instead of individual fluorophores. Although all comparative analyses were conducted under strictly identical and controlled experimental conditions, supporting reliable AF comparison among the different tissues, further confirmation through complementary molecular or biochemical assays would provide additional specificity to the endogenous fluorophore concentration changes and represents an important direction for future investigation. Human applicability of the present approach requires dedicated validation on human LN tissues as present study focuses on MRL-lpr tissues.

## Conclusion

The present manuscript shows the AF spectroscopy and multispectral AF microscopy for the renal tissues using proteinuric, antibody-positive, and young cases. A total of 16 cases were examined in in-vitro mode using a developed micro-spectro-endoscope. The 365 nm excitation wavelength is used for AF microscopy and multispectral microscopy. AF microscopy across medullar, medullar-cortex, and cortex regions revealed higher AF in the cortex for all cases. In the cortex, multispectral analysis showed increased green-band AF in proteinuric and antibody-positive tissues due to elevation in FAD and flavoproteins. The 405 nm excitation wavelength is utilized for simultaneous AF spectroscopy and microscopy. Simultaneous AF spectroscopy and microscopy of the cortex show spectral shifted peaks around ~ 480 nm, ~ 493 nm, and ~ 532 nm in the case of proteinuria, antibody-positive, and young tissues due to changes in concentration of NADH and FAD. Further, a new peak at around ~ 616 nm arises in the antibody-positive case, indicating lipofuscin, which is absent in proteinuria and young tissues. These observations suggest that AF-based methods may offer valuable insights into biochemical changes in renal tissues. While the results are preliminary, further investigation with larger sample sizes could help refine these findings and explore their relevance for renal disease characterization and diagnosis.

## Supplementary Information

Below is the link to the electronic supplementary material.

Supplementary Material 1

## Data Availability

Data underlying the results presented in this paper are not publicly available at this time but may be obtained from the authors upon reasonable request.
