# Supplementary material for: Autofluorescence spectroscopy and multispectral autofluorescence microscopy for characterization of lupus nephritis in renal tissues
Source: Sci Rep. 2026 Jul 2;16:20186. doi: 10.1038/s41598-026-47510-w (PMC13323735; doi:10.1038/s41598-026-47510-w)
Supplement: Supplementary file 1 — Supplementary Material 1 [file 41598_2026_47510_MOESM1_ESM.docx]

**Autofluorescence Spectroscopy and Multispectral Autofluorescence Microscopy for characterization of Lupus Nephritis in Renal Tissues**

Pramila Thapa1*, Vishesh Dubey2, Azeem Ahmad2, Kristin Andreassen Fenton3, Dalip Singh Mehta1, and Balpreet Singh Ahluwalia2#

*1 Bio and Green Photonics Lab, Department of Physics, Indian Institute of Technology Delhi, New Delhi, 110016*

*2 Department of Physics and Technology, UiT the Arctic University of Norway*

*3RNA and Molecular Pathology research group, Department of Medical Biology, Faculty of Health Sciences, UiT the Arctic University of Norway*

[***pramilathapa643@gmail.com**](mailto:*pramilathapa643@gmail.com) **, #** [**balpreet.singh.ahluwalia@uit.no**](mailto:balpreet.singh.ahluwalia@uit.no)

**Supplementary Information**

**S1. Calculation of mean of AF microscopic images**

Figure S1 indicating the analytical workflow used for quantifying autofluorescence (AF) intensity in renal tissue samples from MRL-lpr mice across three groups, proteinuric, antibody-positive, and young. For each group, multiple biological samples were collected. For proteinuric tissue, we have a total of 5 MRL-lpr mice. For antibody-positive and young tissues, we have 5 and 6 MRL-lpr mice. From each sample, several raw AF images were acquired, and the mean intensity value was calculated for each image. To minimize intra-sample variability and ensure that each biological replicate contributed equally, the mean values from all images of a given sample were averaged to produce a single representative value per sample. These sample-wise averages were then used for statistical analysis, including group comparisons.


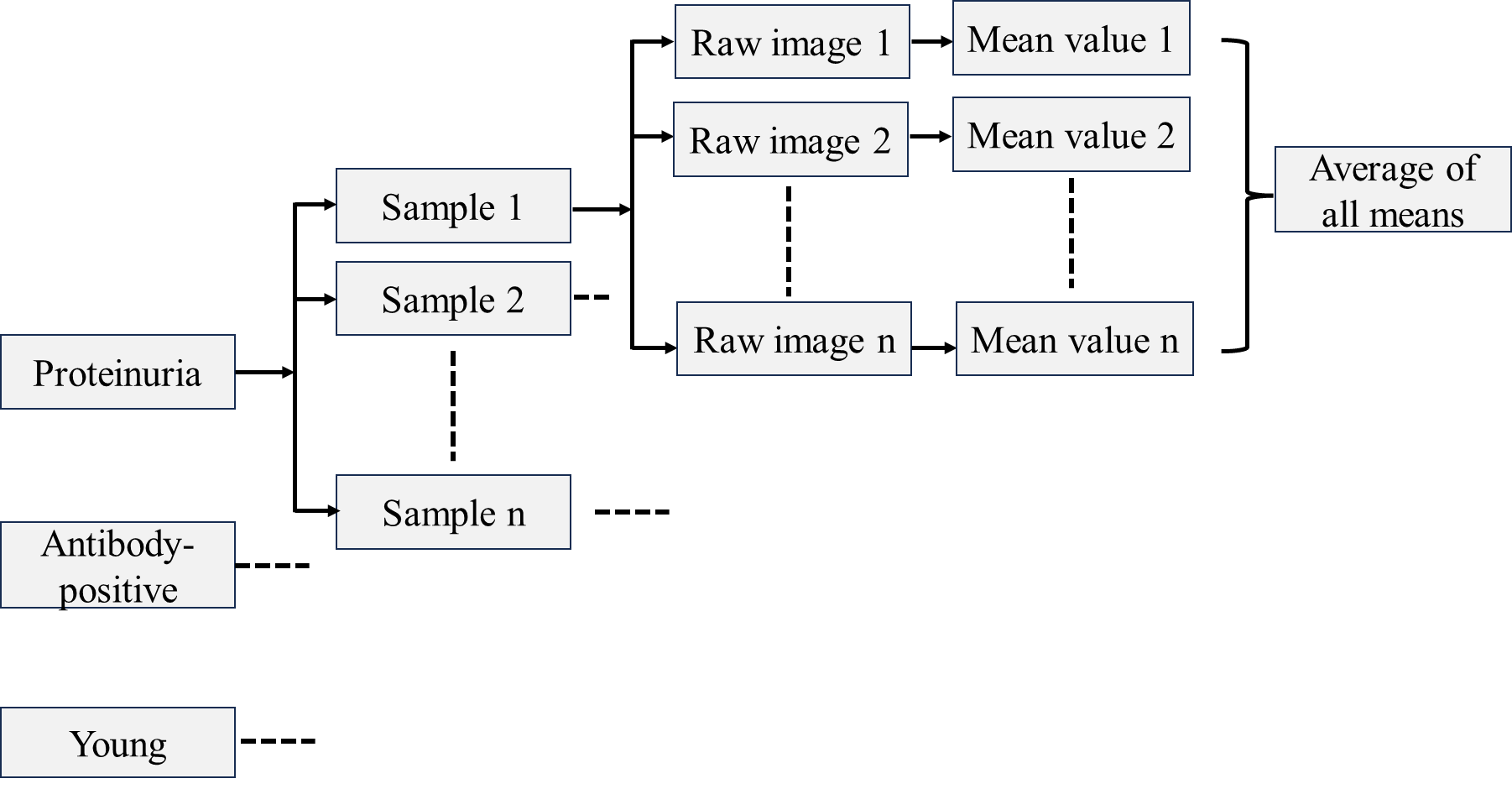


Figure S1. The flowchart is used to calculate average values of AF intensity in microscopic images for each sample of proteinuria, antibody-positive, and young tissues.

**S2. Two-way ANOVA test**

**S2.1 Autofluorescence Microscopy for different Renal regions**

Table S1. Two-way ANOVA test for young, antibody-positive and proteinuric tissues in three different tissue regions, medulla, medullar-cortex and cortex

| Two-way ANOVA | Ordinary |  |  |  |  |
| --- | --- | --- | --- | --- | --- |
| Alpha | 0,05 |  |  |  |  |
|  |  |  |  |  |  |
| Source of Variation | % of total variation | P value | P-value summary | Significant? |  |
| Interaction | 2.61 | 0.0243 | * | Yes |  |
| Row Factor (group) | 18.29 | <0.0001 | **** | Yes |  |
| Column Factor (Tissue region) | 59.24 | <0.0001 | **** | Yes |  |
|  |  |  |  |  |  |
| ANOVA table | SS (Type III) | DF | MS | F (DFn, DFd) | P value |
| Interaction | 550.9 | 4 | 137.71 | F(4, 90) = 2.95 | 0.0243 |
| Row Factor | 3861.5 | 2 | 1930.73 | F(2, 90) = 41.35 | <0.0001 |
| Column Factor | 12502.4 | 2 | 6251.2 | F(2, 90) = 133.88 | <0.0001 |
| Residual | 4202.3 | 90 | 46.69 |  |  |
|  |  |  |  |  |  |
| Data summary |  |  |  |  |  |
| Number of columns (Column Factor) | 3 |  |  |  |  |
| Number of rows (Row Factor) | 3 |  |  |  |  |
| Number of values | 99 |  |  |  |  |

Table S2. Tucky’s Multi-comparison Test for young, antibody-positive and proteinuric tissues in three different tissue regions, medulla, medullar-cortex and cortex

| Within each row, compare columns (simple effects within rows) | | | | | | | | | |
| --- | --- | --- | --- | --- | --- | --- | --- | --- | --- |
|  | | | | | | | | | |
| Number of families: 3 | | | | | | | | | |
| Number of comparisons per family: 3 | | | | | | | | | |
| Alpha: 0.5 | | | | | | | | | |
|  | | | | | | | | | |
| Tucky’s HSD test | | | | | | | | | |
|  |  |  |  |  |  |  |  |  |  |
| Region | **Comparison** | **Mean-Diff** | **CI** | **Below-Threshold** | **p-value** | **Summary** |  |  |  |
| Cortex | Antibody-positive vs. Proteinuric | -13.53 | [-22.67, -4.41] | false | <0.001 | ** |  |  |  |
|  | Antibody-positive vs. Young | 6.05 | [-3.08, 15.18] | false | 0.24 | ns |  |  |  |
|  | Proteinuric vs. Young | 19.59 | [10.46, 28.72] | false | <0.0001 | **** |  |  |  |
| Medulla | Antibody-positive vs. Proteinuric | -2.67 | [-7.82, 2.48] | false | 0.41 | ns |  |  |  |
|  | Antibody-positive vs. Young | 13.24 | [8.10, 18.39] | false | <0.0001 | **** |  |  |  |
|  | Proteinuric vs. Young | 15.91 | [10.77, 21.06] | false | <0.0001 | **** |  |  |  |
| Middle | Antibody-positive vs. Proteinuric | -6.19 | [-12.89, 0.51] | false | 0.074 | ns |  |  |  |
|  | Antibody-positive vs. Young | 4.18 | [-2.51, 10.89] | false | 0.28 | ns |  |  |  |
|  | Proteinuric vs. Young | 10.38 | [3.68, 17.08] | false | <0.001 | ** |  |  |  |
| Test Details | | | | | | | | | |
|  |  |  |  |  |  |  |  |  |  |
| Region | **Comparison** | **Mean1** | **Mean2** | **Meandiff** | **SE** | **N1** | **N2** | **q** | **DF** |
| Cortex | Antibody-positive vs. Proteinuric | 45.45 | 58.99 | -13.53 | 4.47 | 11 | 11 | -3.02 | 30 |
|  | Antibody-positive vs. Young | 45.45 | 39.39 | 6.05 | 4.47 | 11 | 11 | 1.35 | 30 |
|  | Proteinuric vs. Young | 58.99 | 39.39 | 19.59 | 4.47 | 11 | 11 | 4.38 | 30 |
| Medulla | Antibody-positive vs. Proteinuric | 35.22 | 37.89 | -2.67 | 2.52 | 11 | 11 | -1.05 | 30 |
|  | Antibody-positive vs. Young | 35.22 | 21.97 | 13.24 | 2.52 | 11 | 11 | 5.25 | 30 |
|  | Proteinuric vs. Young | 37.89 | 21.97 | 15.91 | 2.52 | 11 | 11 | 6.31 | 30 |
| Middle | Antibody-positive vs. Proteinuric | 19.91 | 26.10 | -6.19 | 3.28 | 11 | 11 | -1.88 | 30 |
|  | Antibody-positive vs. Young | 19.91 | 15.72 | 4.18 | 3.28 | 11 | 11 | 1.27 | 30 |
|  | Proteinuric vs. Young | 26.10 | 15.72 | 10.38 | 3.28 | 11 | 11 | 3.16 | 30 |

**S2.2 Multispectral Autofluorescence Microscopy**

Table S3. Two-way ANOVA test for young, antibody-positive and proteinuric tissues in three different spectral bands, blue, green and red

| Two-way ANOVA | Ordinary |  |  |  |  |
| --- | --- | --- | --- | --- | --- |
| Alpha | 0,05 |  |  |  |  |
|  |  |  |  |  |  |
| Source of Variation | % of total variation | P value | P-value summary | Significant? |  |
| Interaction | \| 5.14 \| \| --- \| | 0.0001 | ** | Yes |  |
| Row Factor (group) | \| 22.84 \| \| --- \| | <0.0001 | **** | Yes |  |
| Column Factor (Tissue region) | \| 21.95 \| \| --- \| | <0.0001 | **** | Yes |  |
|  |  |  |  |  |  |
| ANOVA table | SS (Type III) | DF | MS | F (DFn, DFd) | P value |
| Interaction | 2279.88 | 4 | 569.97 | F(4, 228) = 5.85 | \| 0.0001 \| \| --- \| |
| Row Factor | 10128.29 | 2 | 5064.145 | F(2, 228) = 52.02 | <0.0001 |
| Column Factor | 9733.01 | 2 | 4866.505 | F(2, 228) = 49.99 | <0.0001 |
| Residual | 22195.3 | 228 | 97.34780702 |  |  |
|  |  |  |  |  |  |
| Data summary |  |  |  |  |  |
| Number of columns (Column Factor) | 3 |  |  |  |  |
| Number of rows (Row Factor) | 3 |  |  |  |  |
| Number of values | 237 |  |  |  |  |

**Table S4. Tucky’s Multi-comparison Test for young, antibody-positive and proteinuric tissues in three different filters, blue, green and red**

| Within each row, compare columns (simple effects within rows) | | | | | | | | | |
| --- | --- | --- | --- | --- | --- | --- | --- | --- | --- |
|  | | | | | | | | | |
| Number of families: 3 | | | | | | | | | |
| Number of comparisons per family: 3 | | | | | | | | | |
| Alpha: 0.5 | | | | | | | | | |
|  | | | | | | | | | |
| Tucky’s HSD test | | | | | | | | | |
|  |  |  |  |  |  |  |  |  |  |
| Region | Comparison | MeanDiff | CI | BelowThreshold | PValue | Summary |  |  |  |
| blue | antibody-positive vs. proteinuric | 2.30 | -3.3438 | 7.9513 | 0.5946 | ns |  |  |  |
| blue | antibody-positive vs. young | -20.01 | -25.3635 | -14.648 | <0.0001 | *** |  |  |  |
| blue | proteinuric vs. young | -22.31 | -27.6672 | -16.9518 | <0.0001 | *** |  |  |  |
| green | antibody-positive vs. proteinuric | 2.86 | -5.0239 | 10.7547 | 0.6627 | ns |  |  |  |
| green | antibody-positive vs. young | -8.41 | -15.5392 | -1.2878 | 0.0165 | * |  |  |  |
| green | proteinuric vs. young | -11.28 | -18.4046 | -4.1532 | 0.0009 | *** |  |  |  |
| red | antibody-positive vs. proteinuric | -6.32 | -12.8683 | 0.2367 | 0.0612 | ns |  |  |  |
| red | antibody-positive vs. young | -12.54 | -19.0263 | -6.0531 | 0 | *** |  |  |  |
| red | proteinuric vs. young | -6.22 | -12.7105 | 0.2628 | 0.0627 | ns |  |  |  |
|  | | | | | | | | | |
| Test Details | | | | | | | | | |
|  |  |  |  |  |  |  |  |  |  |
| Region | Comparison | Mean1 | Mean2 | MeanDiff | SE | N1 | N2 | q | **DF** |
| blue | antibody-positive vs. proteinuric | 46.84875 | 49.1525 | 2.30 | 1.57852598 | 24 | 24 | -1.45 | 75 |
| blue | antibody-positive vs. young | 46.84875 | 26.843 | -20.01 | 1.57852598 | 24 | 30 | 12.67 | 75 |
| blue | proteinuric vs. young | 49.1525 | 26.843 | -22.31 | 1.57852598 | 24 | 30 | 14.13 | 75 |
| green | antibody-positive vs. proteinuric | 53.90375 | 56.76916667 | 2.86 | 1.686890343 | 24 | 24 | -1.69 | 83 |
| green | antibody-positive vs. young | 53.90375 | 45.49026316 | -8.41 | 1.686890343 | 24 | 38 | 4.99 | 83 |
| green | proteinuric vs. young | 56.76916667 | 45.49026316 | -11.28 | 1.686890343 | 24 | 38 | 6.69 | 83 |
| red | antibody-positive vs. proteinuric | 44.1225 | 37.80666667 | -6.32 | 1.770810345 | 24 | 24 | 3.57 | 70 |
| red | antibody-positive vs. young | 44.1225 | 31.5828 | -12.54 | 1.770810345 | 24 | 25 | 7.08 | 70 |
| red | proteinuric vs. young | 37.80666667 | 31.5828 | -6.22 | 1.770810345 | 24 | 25 | 3.51 | 70 |

**S2. Autofluorescence spectra for long pass filter (>500 nm)**

We have further recorded AF spectra of all tissues using >500 nm long pass filter. Figure S2presents normalized AF spectra from all groups, young (green), antibody-positive (blue), and proteinuric (red) tissues. Each spectra represents the mean AF intensity for cases, while the shaded envelopes show standard deviation. Young tissue shows a peak emission at 559 nm with a FWHM of 83 nm and an AUC of 93.5. Antibody-positive tissue demonstrates a red-shifted peak at 595 nm, a FWHM of 85 nm, and a lower AUC of 76.1, suggesting altered fluorophore concentration and reduced overall emission. Proteinuric tissue peaks at 560 nm with the broadest FWHM (94 nm) and the highest AUC (97.0), reflecting enhanced spectral spread and increased accumulation of auto fluorescent compounds, likely associated with advanced disease progression.

FigureS2. Normalized AF spectra of renal cortex tissues from young (green), antibody-positive (blue), and proteinuric (red) groups. Solid lines represent mean AF intensities, while shaded regions indicate standard deviation.
